# Predictors of Information Technology Integration in Secondary Schools: Evidence from a Large Scale Study of More than 30,000 Students

**DOI:** 10.1371/journal.pone.0168547

**Published:** 2016-12-20

**Authors:** Khe Foon Hew, Cheng Yong Tan

**Affiliations:** Faculty of Education, The University of Hong Kong, Hong Kong, Hong Kong SAR; University of Westminster, UNITED KINGDOM

## Abstract

The present study examined the predictors of information technology (IT) integration in secondary school mathematics lessons. The predictors pertained to IT resource availability in schools, school contextual/institutional variables, accountability pressure faced by schools, subject culture in mathematics, and mathematics teachers’ pedagogical beliefs and practices. Data from 32,256 secondary school students from 2,519 schools in 16 developed economies who participated in the Program for International Student Assessment (PISA) 2012 were analyzed using hierarchical linear modeling (HLM). Results showed that after controlling for student-level (gender, prior academic achievement and socioeconomic status) and school-level (class size, number of mathematics teachers) variables, students in schools with more computers per student, with more IT resources, with higher levels of IT curricular expectations, with an explicit policy on the use of IT in mathematics, whose teachers believed in student-centered teaching-learning, and whose teachers provided more problem-solving activities in class reported higher levels of IT integration. On the other hand, students who studied in schools with more positive teacher-related school learning climate, and with more academically demanding parents reported lower levels of IT integration. Student-related school learning climate, principal leadership behaviors, schools’ public posting of achievement data, tracking of school’s achievement data by administrative authorities, and pedagogical and curricular differentiation in mathematics lessons were not related to levels of IT integration. Put together, the predictors explained a total of 15.90% of the school-level variance in levels of IT integration. In particular, school IT resource availability, and mathematics teachers’ pedagogical beliefs and practices stood out as the most important determinants of IT integration in mathematics lessons.

## Introduction

In the recent decades, many schools have jumped on the bandwagon to exploit advances in information technology (IT) in the endeavor to enhance students’ learning and achievement. This societal trajectory is corroborated by the proliferation of studies examining the impact of IT on achievement [1, 2, 3]. However, there is little evidence to-date to unequivocally support or refute the rhetoric that IT can enhance student achievement. For example, Cheung and Slavin’s [1] meta-analysis of 74 studies showed that educational technology applications were, on average, modestly related with mathematics learning outcomes. However, recently published analysis of PISA 2012 data [4] showed that access to IT resources was not associated with enhanced mathematics achievement. Even experimental studies showed mixed evidence for the contribution of IT to student achievement [5, 6].

Instead of asking whether school IT resources have a conclusive positive relationship with student achievement, it may be more fruitful to examine why and how these resources are used in teaching-learning in schools in the first place. Put another way, two schools may have different compelling reasons for integrating IT into teaching-learning practices. These reasons may then moderate the effects of IT on student achievement. Indeed, earlier studies have suggested that IT *per se* does not necessarily translate to optimal usage; instead IT integration in schools represents a more useful indicator of IT impact [7, 8]. This paradigm shift in thinking has resulted in many scholars attempting to “unpack” what IT integration in teaching-learning means in the school context. Some scholars relate IT integration to teachers using technology for instructional preparation [9], instructional delivery [10], or simply to enhance the effectiveness of implementing teachers’ usual activities [11]. More recently, however, scholars have shifted the focus from teachers’ work to students’ learning. For example, Cuban, Kirkpatrick, and Peck [12] emphasize the different levels of IT usage by students in their class learning, while other researchers understand IT as students learning higher-order competencies and skills such as critical and creative thinking, problem-solving, and social communication [13].

Given the potential of IT integration on students’ achievement, researchers quite understandably shift their attention to examine what IT-related variables predict effective IT integration in teaching-learning in schools [14, 15, 16]. However, students’ learning is a complex multi-factorial process affected by student-teacher proximal interactions and other indirect contextual factors in the school ecology [16, 17]. Therefore, other school and teacher variables that are not directly related to IT may be equally important in determining the degree of IT integration in schools. The paucity of studies examining non-IT related variables represent an important gap to be addressed.

Therefore, the present study aims to (a) examine different IT and non-IT related predictors of the levels of IT integration in schools, and (b) identify which sets of predictors are more important than others in predicting IT integration. It takes advantage of a large dataset on IT integration involving fifteen-year-old students’ involvement in mathematics lessons (and not merely teacher-centered presentations), and many predictor variables (namely, IT resource availability in schools, school contextual/institutional variables, accountability pressure faced by schools, subject culture in mathematics, and mathematics teachers’ pedagogical beliefs and practices) using the hierarchical linear modeling (HLM) approach. The availability of the multitudinous variables allows for the examination of the independent effects of each predictor after controlling for other predictor and student- and school-level control variables (e.g., students’ prior academic achievement, number of mathematics teachers). This approach also represents a significant methodological improvement over isolated studies focusing on specific variables [16, 18] (O’Dwyer, Russell, & Bebel, 2004; Zhao & Frank, 2003). More specifically, it addresses Zhao and Frank’s [16] concern that

“In summary, previous research has resulted in a large, almost inexhaustible, list of factors that may affect the uses of technology in schools. However, these factors are often examined in isolation from each other or from the system in which they interact.” (pp. 809–810).

Furthermore, this approach contrasts with meta-analytic studies that attempt to compare the effectiveness of different predictors obtained from various individual studies that may not necessarily be comparable in methodological design [19]. At the same time, the use of HLM recognizes the structure of the data (students nested in schools), and allows for the degree of IT integration to be appropriately examined at the school (vis-a-vis student) level [20]. Although the participants of PISA 2012 come from 68 different economies, the present study focuses on only those from the Organization of Economic Cooperation and Development (OECD) economies. This decision is informed by previous evidence showing that the quality of schools may vary as function of national economic development [21], thereby enabling us to limit the sample to more comparable schools, while overcoming the problem of range restriction in variables common in small-scale studies [22].

## Background

We begin with a clarification of the key term–IT integration. In the present study, we adapt the description used by Hew and Brush [14] to define IT integration as the use of computing devices such as desktop computers, laptops, tablets, software, or Internet for educational purposes. More specifically, this study considers IT integration as the use of technology as a tool for learning mathematics. It includes participants using devices and software to extend their cognitive abilities to understand mathematics concepts, or solve problems such as drawing graphs of functions, constructing geometric figures, entering data in spreadsheets, drawing histograms, and determining how graphs change according to different parameters.

Previous research has found that using IT as a learning tool (such as the aforementioned examples) can improve student mathematics learning [1, 2]. For example, Li and Ma [2] meta-analyzed more than 40 experimental or quasi-experimental articles that employed IT for learning purposes, and used mathematics achievement as outcome. The results of their analysis suggested that using IT as a learning tool (e.g., spreadsheet, Geometer’s Sketchpad) positively impacted mathematics achievement of students.

Although studies have shown that use of IT as a learning tool can help student learning of mathematics, IT use is typically affected by certain school- and teacher-level factors. A review of the relevant studies suggests a list of factors, which can be parsimoniously categorized into the following dichotomy: first- and second-order factors.

First-order factors are elements external to teachers [23]. Examples of the *most commonly* reported first-order factors include IT infrastructure such as availability and access to hardware and software [14, 24, 25, 26], institutional support such as management encouragement [18, 27], technical support [28], subject and assessment culture [11], and shared school IT vision and policy.

In contrast, second-order factors are elements intrinsic to teachers [23]. The most commonly reported second-order factors are teacher professional development such as computer knowledge or skill training [24, 29, 30], and teacher beliefs [31, 32, 33]. Ertmer [34] who examine teacher beliefs about teaching and learning, consider these beliefs pedagogical and viewed them as the “final frontier” (p. 25) in the quest for IT integration due to the stronger influence of beliefs as opposed to computer knowledge or skill in predicting teacher behavior. Two prototypical ideologies are commonly discussed–teacher-centered or learner-centered belief [31, 32, 33, 35, 36]. Studies have found that teachers with learner-centered beliefs were more likely to have a positive attitude (computer liking), and motivation to conduct IT integrated lessons [31, 33].

## Limitations of past relational studies

Although past relational studies have suggested various predictors of IT integration, the results must be interpreted with caution. First, previous research on IT integration is often limited to the study of teacher-level factors [29, 37]. There are relatively few empirical studies that examine school related or subject culture factors in secondary school mathematics lessons.

Second, there is no control for variables such as students’ prior academic achievement and the number of teachers. Students’ prior academic achievement is an important variable in learning [38]. It is possible that teachers conduct more IT integrated lessons as a remedial or compensatory strategy for students with lower prior achievement compared to students with higher achievement. Likewise, the number of teachers may affect the frequency of IT integration because schools with more teachers might have difficulty in scheduling the computer labs for students to use. Hence students’ prior academic achievement and the availability of qualified teachers may be confounding variables if they are not controlled.

The third limitation is that past studies exhibit a heavy reliance on linear regression analyses. Richter [39] note that such analyses are incapable of handling complex data structures associated with students being nested in schools [40]. The fourth limitation is the tendency of studies to rely on teachers’ self-rating of the frequency or extent to which they use IT [15, 41, 42]. Teachers’ self-report data may be compromised by social desirability bias [43]. Finally, many studies tend to group different categories of technology use as “IT integration”. For example, Tondeur and colleagues [37] included the use of computers to develop students’ technical skills (e.g., how to use the keyboard), in addition to use of computers as a learning tool as IT integration.

In this study we address the aforementioned limitations. The present study is unique in that we specifically examined IT integration as the use of IT as a learning tool for secondary school mathematics, rather than computer skills. IT integration in mathematics lessons is examined in light of the importance of mathematical and scientific competencies in knowledge-based economies [44]. Data from 32,256 students from 16 developed countries were analyzed, thereby diminishing the limitations of country-specific results. We controlled for students’ academic achievement and the shortage of teachers, among other variables. We also examined the possible effects of school-level variables such as students’ behavioral climate (e.g., students skipping classes, truancy), principal-related activities (e.g., promoting evidence-based teaching practices, evaluating classroom instruction), the accountability pressure schools faced on students’ academic achievement, and mathematics subject culture. These variables, to our best knowledge, have not been examined in past studies on IT integration in secondary school mathematics. To manage the nested data, the present study employed HLM to address the possible correlation in achievement scores of students belonging to the same school and to partition achievement into between-school (the appropriate level for the purposes of the present study) as opposed to within-school variance [20]. Finally, the measurement of IT integration using students’ reported data, instead of teachers’, helped circumvent the problems of possible teachers’ inaccurate responses or social desirability bias [43].

## Material and Method

### Participants

Participants were students and school principals who participated in PISA 2012 conducted by OECD. PISA 2012 measured the proficiency of approximately 500,000 15-year-old students from 68 economies (OECD and non-OECD members) in applying their knowledge and skills learned in reading, mathematics, and science to authentic problems. However, only the data from OECD economies were examined in the present study. Cases with missing data for any of the variables investigated were excluded. This resulted in a final sample of 32,256 students from 2,519 schools in 16 OECD economies available for analysis. These economies comprised Australia, Austria, Belgium, Switzerland, Chile, Denmark, Hungary, Ireland, Israel, Italy, Korea, Mexico, New Zealand, Portugal, Slovak Republic, and Sweden.

### Measures

Available data on the following variables from the PISA 2012 dataset were used in the analysis (Table 1). In the following paragraphs, we describe each variable in greater detail.

**Table 1:** Description, Mean, and Standard Deviation of Variables.

Category	Variables and description	*M(SD)*
IT integration	• *Integration–*using IT as a learning tool (Inan & Lowther, 2010; Tondeur et al., 2007). This includes using desktop computers, laptops or tablets to draw graphs of functions, enter data in spreadsheets, and find out how graphs changed depending on parameters.	1.42(0.59)
IT infrastructure	• *CompPerStu–*student-to-computer ratio	0.66(1.00)
	• *ITMain*–availability of various IT resources (e.g., desktop computers, laptops, tablet computers, Internet connection) for students to use	2.49(0.57)
Institution	• *Curriculum*–IT curricular expectations such as the proportion of school work whereby students are expected to use the Internet	2.64(0.91)
	• *ClimateTr*–teacher climate such as staff resisting change, teachers being ill-prepared for classes	3.02(0.52)
	• *ClimateStu–*student climate such as students skipping classes, arriving late for school	2.89(0.57)
	*Principal behaviors*	
	• *Academic–*principals promoting evidence-based teaching practices, using students’ performance results to develop school goals	3.67(1.09)
	• *React*–principals solving problems such as taking initiative to discuss classroom challenges with teachers	(1.10)
	• *Monitor–*principals monitoring and evaluating classroom instruction and staff performance	2.91(1.10)
Accountability	• *ParentExp–the degree of parental pressure to achieve high academic standards*	1.93(0.70)
	• *DataPublic–whether the school posted student achievement publicly*	0.44(0.50)
	• *DataTracked—whether the school tracked student achievement*	0.69(0.46)
Math subject culture	• *Policy–whether a school-wide policy on how to use IT in mathematics instruction was present*	0.32(0.47)
	• *Differentiate–the degree of pedagogical and curricular differentiation in mathematics learning such as whether students are grouped by ability*	1.82(0.54)
Teacher belief and practice	• *PedagogyBelief—teachers’ conceptions of student-centered mathematics teaching and learning*	2.85(0.52)
	• *ProbSolve–frequency of problem solving activities in mathematics lessons*	2.85(0.74)
	• *ProbGive–frequency of teacher presenting novel problems in mathematics lessons such as giving problems without obvious solutions*	2.46(0.71)
Student-level control	• *Male–whether student was male*	0.48(0.50)
	• *Repeat–whether student had ever repeated a grade*	3.06(0.31)
	• *MoEdu–mother’s level of education as a proxy of students’ social economic status*	4.16(1.05)
School-level control	• *ClassSize–the average class size for the modal grade for 15-year old students in the school*	4.26(2.13)
	• *InFTTr–ln (number of full-time mathematics teachers)*	1.76(0.80)
	• *InPTTr–ln (number of part-time mathematics teachers)*	0.82(0.76)

#### IT integration

IT integration was measured using students’ responses to seven questions asking them if computers were used in mathematics lessons (1 = *No*, 2 = *Yes*, *but only with teacher demonstration*, 3 = *Yes*, *with students using computers*). The topics comprised drawing graphs of functions, performing calculations, constructing geometric figures, entering data in spreadsheets, rewriting algebraic expressions and solving equations, drawing histograms, and finding out how graphs changed depending on parameters. The responses were subjected to exploratory factor analysis (EFA) (*varimax* rotation) using principal component analysis. Results showed that the seven items could be explained by one factor (*eigenvalue* = 4.55) explaining 65.03% of the variance. Therefore, a scale was constructed by averaging the responses to these items and named Integration (α = .91). A greater value for this variable is viewed as representing a higher level of IT integration.

#### IT resources

Two variables measured the availability of IT resources in schools, namely student-computer ratio, and the availability of different types of IT resources for students to use in school. First, principals provided information on the (a) total number of computers available for educational purposes for the modal grade corresponding to fifteen-year-old students, and (b) total number of these students in their schools in the School Questionnaire. The student-computer ratio in each school (CompPerStu) was computed by dividing (a) by (b).

Next, students responded to seven questions asking if various IT resources were *available* for them to use in school (1 = *No*, 2 = *Yes*, *but the student did not use it*, 3 = *Yes*, *and the student used it*). These resources comprised desktop computers, portable laptop or notebooks, tablet computers, Internet connection, printers, USB (memory) sticks, and e-book readers. The responses were subjected to EFA (*varimax* rotation) using principal component analysis. Results from the third round of EFA, after deleting two items that exhibited cross-loading on more than one factor, showed that the items could be summarized by one factor (*eigenvalue* = 1.90) explaining 37.95% of the variance. A scale (ITMain; α = .71) was constructed by averaging the responses to the three items that loaded on this factor (pertaining to desktop computers, Internet connection, and printers).

#### Institution

Six institutional variables pertaining to IT curricular expectations, school climate, and principal leadership behaviors were measured. First, a variable (Curriculum) was constructed by averaging principals’ responses to three questions asking about the proportion of schoolwork (work during lessons, homework, and assignments/projects) whereby students were expected to access the Internet (1 = *<10%*, 2 = *10–25%*, 3 = *26–50%*, 4 = *51–75%*, 5 = *>75%*).

Next, principals’ responses to 19 questions asking if various climate-related phenomena impeded students’ learning in their schools (e.g., student truancy, staff resisting change) were coded as follows: 1 = *A lot*, 2 = *To some extent*, 3 = *Very little*, 4 = *Not at all*). The responses were subjected to EFA (*varimax* rotation) using principal component analysis. Results from the second round of EFA, after deleting items that exhibited cross-loading on more than one factor, showed that the items could be summarized by two factors explaining a total of 48.82% of the variance. Five items measuring staff resisting change, teachers not meeting individual students’ needs, teachers being too strict with students, teachers being ill-prepared for classes, and students not being encouraged to achieve their full potential loaded on the first factor (*eigenvalue* = 2.79; 25.32% of variance explained). A scale (ClimateTr) was constructed by averaging the responses to these teacher-related items that loaded on this factor (α = .80). Four items measuring students skipping classes, truancy, arriving late for school, and not attending compulsory school events loaded on the second factor (*eigenvalue* = 2.59; 23.50% of variance explained). A scale (ClimateStu) was constructed by averaging the responses to these student-related items that loaded on this factor (α = .81). A greater value for these climate variables represented a more positive school learning climate.

Principals also responded to 22 questions asking about the frequency of different activities they were involved in (e.g., “I work to enhance the school’s reputation in the community”) using a six-point scale (1 = *Did not occur*, 2 = *1–2 times yearly*, 3 = *3–5 times yearly*, 4 = *Monthly*, 5 = *Weekly*, 6 = *More than once a week*). The responses were subjected to EFA (*varimax* rotation) using principal component analysis. Results from the third round of EFA, after deleting items that exhibited cross-loadings, showed the items could be summarized by three factors explaining a total of 66.05% of the variance. The first factor on principals’ instructional activity(*eigenvalue* = 3.00; 27.26% of variance explained) comprised five items measuring principals ensuring the alignment of teachers’ professional development with school teaching goals, promoting evidence-based teaching practices, using students’ performance results to develop school goals, ensuring that teachers worked according to school goals, and enhancing school’s reputation in the community. The second factor on principals resolving classroom problems (*eigenvalue* = 2.23; 20.23% of variance explained) comprised three items measuring principals solving classroom problems together with teachers, taking initiative to discuss classroom problems, and paying attention to disruptive classroom behaviors. The third factor on principals’ monitoring (*eigenvalue* = 2.04; 18.56% of variance explained) comprised three items measuring principals reviewing student work when evaluating classroom instruction, conducting informal classroom observations regularly, and evaluating staff performance. Three scales corresponding to the three factors were constructed by averaging the responses to items loading on each of the factors (Academic, α = .84; React, α = .81; Monitor, α = .74 respectively).

#### Accountability

Three variables were constructed to measure the accountability pressure schools faced on students’ academic achievement. First, a variable (ParentExp) was constructed to measure principals’ responses to a question asking about the degree of parental pressure schools encountered to achieve high academic standards (1 = *Largely absent*, 2 = *Pressure from minority of parents*, 3 = *Constant pressure from many parents*). Next, two dichotomous variables were constructed to measure the existence of two principal-reported school practices (1 = *Yes*, 0 = *No*): posting achievement data publicly (DataPublic), and tracking of achievement data by administrative authorities (DataTracked).

#### Mathematics subject culture

Subject culture refers to the ‘‘general set of institutionalized practices and expectations which have grown up around a particular school subject” (Goodson & Mangan, 1995, p. 614). In the present study, two variables measured the expectations and norms pertaining to mathematics in schools. First, a dichotomous variable (Policy) was constructed to measure the presence of a school policy that describes the expected ways in which technology is used for learning mathematics (1 = *Yes*, 0 = *No*).

Next, three questions were asked about the degree of pedagogical and curricular differentiation in mathematics (i.e., if students in mathematics classes studied similar content but at different levels of difficulty, if students studied different content of mathematics topics at different difficulty levels, and if students were grouped by ability within mathematics classes using a three-point scale (1 = *Not for any classes*, 2 = *For some classes*, 3 = *For all classes*). The responses were subjected to EFA (*varimax* rotation) using principal component analysis. Results showed that the items could be summarized by one factor (*eigenvalue* = 1.60) explaining 53.46% of the variance. A scale (Differentiate) was constructed by averaging the responses to the three items (α = .56).

#### Mathematics teacher beliefs and practices

Three variables measured mathematics teachers’ pedagogical beliefs and practices. First, three questions were asked about mathematics teachers’ conceptions of student-centered teaching and learning such as adapting standards to students’ needs using a four-point scale (1 = *Strongly disagree*, 2 = *Disagree*, 3 = *Agree*, 4 = *Strongly agree*). The responses were subjected to EFA (*varimax* rotation) using principal component analysis. Results showed that the items could be summarized by one factor (*eigenvalue* = 1.68) explaining 55.88% of the variance. A scale (PedagogyBelief) was constructed by averaging the responses to the items (α = .60).

Next, students responded to nine questions asking about the frequency of problem-solving activities in their mathematics classes (e.g., teachers asking questions that made students reflect on problems, teachers asking students to explain how they solved problems) using a four-point scale (1 = *Never or rarely*, 2 = *Sometimes*, 3 = *Often*, 4 = *Always or almost always*). The responses were subjected to EFA (*varimax* rotation) using principal component analysis. Results of the second round of EFA showed that, after deleting items that cross-loaded on more than one factor, the items could be summarized by two factors explaining a total of 60.99% of the variance. The first factor (*eigenvalue* = 1.85; 30.77% variance explained) comprised three items measuring teachers facilitating students’ problem-solving capacities (namely, helping students to learn from their mistakes, asking students to explain their solutions, and getting students to apply their learning to new contexts). The second factor (*eigenvalue* = 1.81; 30.22% variance explained) comprised three items measuring teachers presenting novel problems to students (namely, presenting problems without obvious solutions, giving problems that required students to think for a while, and asking students to decide on their own procedures for solving problems). Two scales were constructed by averaging the responses to the items for each factor (ProbSolve, α = .68; ProbGive, α = .66 respectively).

#### Controls

Three student-level and three school-level variables were used as controls in the analysis. The student-level controls comprised students’ gender, prior academic achievement, and SES. First, a dummy variable, Male, was coded as 0 for female and 1 for male students. Students also responded to three items indicating whether they had ever repeated a grade at the primary, lower secondary, and upper secondary level using a three-point scale (1 = *No*, *never;* 2 = *Yes*, *once;* 3 = *Yes*, *twice or more*). These responses were added up to form a measure of students’ prior academic achievement (Repeat), with higher values indicating lower levels of students’ prior academic achievement.

Three indicators (parents’ education, occupation, and income) have been used to measure SES in the literature. There is evidence that these indicators are highly correlated with each other, with more educated parents enjoying work of higher occupational status and earning a higher income. In the present study, parents’ education was used to measure students’ familial SES. More specifically, mothers’ as opposed to fathers’ education was used because prior research showed that it was a more predictive variable of student achievement (Chiu & Khoo, 2005). Therefore, a variable (MoEdu) measuring mothers’ responses to items measuring their highest level of schooling completed (1 = *Did not complete primary education*, 2 = *Completed primary education*, 3 = *Completed lower secondary education*, 4 = *Completed upper secondary education that provided direct access to labor markets or to non-university tertiary education*, 5 = *Completed upper secondary education that provided access to university level or non-university tertiary education*) was included in the analysis.

The three school-level controls comprised the average class size and number of mathematics teachers in schools. First, ClassSize measured the average class size (principal-reported) for the modal grade for fifteen-year-old students in schools (1 = *≤15 students*, 2 = *16–20 students*, 3 = *21–25 students*, 4 = *26–30 students*, 5 = *31–35 students*, 6 = *36–40 students*, 7 = *41–45 students*, 8 = *46–50 students*, 9 = *>50 students*). *ln*FTTr and *ln*PTTr were obtained by applying the logarithmic transformation to the data on number of full-time and part-time mathematics teachers in each school (principal-reported) to address problems of violations of normality assumption in the two latter variables.

The correlation between the variables are summarized in Table 2.

**Table 2:** Summary of Inter-correlations for Variables.

Variables	1	2	3	4	5	6	7	8	9	10	11	12	13	14	15	16	17	18	19
1. Integration	-	.01*	-.06**	.01*	-.03**	-.01*	.04**	.10**	.05**	-.02**	-.03**	.02**	.02**	.00	-.02**	.01	.03**	.12**	.13**
2. Repeat		-	-.06**	-.09**	-.01*	.01	.01	-.02**	-.07**	.01*	-.02**	-.04**	.01*	-.02**	-.07**	-.01*	-.02**	-.01*	-.01
3. MoEdu			-	-.21**	.18**	-.05**	.05**	.06**	-.00	.09**	.14**	.03**	-.03**	.01	.11**	-.01	-.05**	-.01	.01
4. ClassSize				-	-.22**	.15**	-.19**	-.16**	.15**	-.10**	-.06**	.02**	-.02**	.12**	.03**	.02**	.16**	.01*	-.02**
5. *ln*FTTr					-	.02**	.03**	.06**	-.03**	.05**	.08**	.05**	.03**	-.02**	.09**	.04**	-.11**	-.01	.01*
6. *ln*PTTr						-	-.03**	.01*	-.04**	.02**	.01	-.09**	-.07**	.02**	-.03**	.05**	-.03**	.03**	.03**
7. CompPerStu							-	.18**	.03**	.05**	-.01	.12**	-.01	.06**	.04**	.07**	.01	.02**	.03**
8. ITMain								-	.02**	.06**	.02**	.04**	-.03**	-.01	.02**	.04**	-.00	.07**	.06**
9. Curriculum									-	-.04**	.00	.14**	.05**	.12**	.15**	.05**	.10**	.00	-.00
10. ClimateTr										-	.40**	.03**	-.05**	.03**	-.02**	-.03**	.33**	.02**	.03**
11. ClimateStu											-	-.03**	-.09**	-.01*	.09**	-.08**	.06**	.03**	.04**
12. Academic												-	.49**	.40**	.22**	.12**	.12**	.04**	.03**
13. React													-	.34**	.05**	.03**	.06**	.01	-.00
14. Monitor														-	.14**	.11**	.12**	-.00	.00
15. ParentExp															-	.15**	.06**	.03**	.03**
16. Differentiate																-	.14**	.04**	.01
17. PedagogyBelief																	-	.04**	-.00
18. ProbSolve																		-	.44**
19. ProbGive																			-

*Note*. For all variables, higher scores are indicative of more extreme responses in the direction of the construct assessed.

*p < .05.

**p < .01.

### Procedure

PISA 2012 involved all 34 OECD and 31 partner economies (OECD, 2013). All participating economies followed standardized procedures outlined in the technical standards and manuals provided. In addition, students, school principals, and parents (in some economies) completed related questionnaires pertaining to student learning.

## Results

Models predicting IT integration in mathematics lesson were examined for different sets of variables in the following sequence: controls, IT resource availability, institutional variables, assessment pressure, mathematics subject culture, and mathematics teacher beliefs and practices. Centered independent variables were used in all the HLM models to enhance the interpretability of the results and to minimize the problem of multi-collinearity.

HLM results (Table 3) for the null model (Model 1) showed that 85.29% and 14.71% of the variance in IT integration in mathematics lessons occurred at levels 1 (within-school) and 2 (between-school) respectively. These results supported the use of HLM models which took into account the non-independence of IT integration experienced by students who belonged to the same school.

**Table 3:** Fixed Effects Estimates (Top) and Variance-Covariance Estimates (Bottom) for Models of the Predictors of IT Integration in Mathematics Lessons.

Parameter	Model 1	Model 2	Model 3	Model 4	Model 5	Model 6	Model 7
	Fixed effects						
Intercept	1.44**(0.01)	1.48**(0.01)	1.48**(0.01)	1.48**(0.01)	1.48**(0.01)	1.50**(0.01)	1.50**(0.01)
Male		0.09**(0.01)	0.09**(0.01)	0.09**(0.01)	0.09**(0.01)	0.09**(0.01)	0.08**(0.01)
Repeat		0.03**(0.01)	0.04**(0.01)	0.04**(0.01)	0.04**(0.01)	0.04**(0.01)	0.04**(0.01)
MoEdu		-0.01*(0.00)	-0.01**(0.00)	-0.01**(0.00)	-0.01**(0.00)	-0.01**(0.00)	-0.01**(0.00)
ClassSize		0.00(0.00)	0.01*(0.00)	0.00(0.00)	0.00(0.00)	0.00(0.00)	0.00(0.00)
*ln*FTTr		-0.02*(0.01)	-0.02**(0.01)	-0.02**(0.01)	-0.02*(0.01)	-0.02*(0.01)	-0.01*(0.01)
*ln*PTTr		-0.02**(0.01)	-0.02**(0.01)	-0.02*(0.01)	-0.02**(0.01)	-0.02**(0.01)	-0.02**(0.01)
CompPerStu			0.01**(0.01)	0.01*(0.01)	0.01*(0.01)	0.01*(0.01)	0.01*(0.00)
ITMain			0.08**(0.01)	0.08**0.01)	0.08**(0.01)	0.08**(0.01)	0.07**(0.01)
Curriculum				0.03**(0.01)	0.03**(0.01)	0.03**(0.01)	0.03**(0.01)
ClimateTr				-0.01(0.01)	-0.01(0.01)	-0.02(0.01)	-0.03*(0.01)
ClimateStu				-0.01(0.01)	-0.01(0.01)	-0.01(0.01)	-0.01(0.01)
Academic				0.01(0.01)	0.01(0.01)	0.01(0.01)	0.01(0.01)
React				0.01(0.01)	0.00(0.01)	0.00(0.01)	0.00(0.01)
Monitor				-0.01(0.01)	-0.01(0.01)	-0.01(0.01)	-0.01(0.01)
ParentExp					-0.02*(0.01)	-0.02*(0.01)	-0.02**(0.01)
DataPublic					-0.01(0.01)	-0.01(0.01)	-0.01(0.01)
DataTracked					0.02(0.01)	0.02(0.01)	0.01(0.01)
Policy						0.04**(0.01)	0.04**(0.01)
Differentiate						0.01(0.01)	0.00(0.01)
PedagogyBelief							0.03*(0.01)
ProbSolve							0.05**(0.00)
ProbGive							0.07**(0.00)
	Random parameters						
Level 1 intercept	0.3012**(0.00)	0.2996**(0.00)	0.2989**(0.00)	0.2989**(0.00)	0.2989**(0.00)	0.2989**(0.00)	0.2945**(0.00)
Level 2 intercept	0.0519**(0.00)	0.0503**(0.00)	0.0473**(0.00)	0.0465**(0.00)	0.0463**(0.00)	0.0460**(0.00)	0.0437**(0.00)
% Level 1 variance	85.29	85.62	86.35	86.55	86.59	86.66	87.08
% Level 2 variance	14.71	14.38	13.65	13.45	13.41	13.34	12.92
% Reduction in Level 1 variance when compared to Model 1		0.55	0.78	0.79	0.79	0.79	2.24
% Reduction in Level 2 variance when compared to Model 1		3.12	9.03	10.56	10.92	11.45	15.90
-2 Restricted log likelihood	55,619.14	55,443.36	55,288.30	55,301.93	55,313.29	55,316.23	54,809.06

*Note*. Standard errors in parentheses.

* *p* < .05.

** *p* < .01.

When the various control variables were included in the model (Model 2), results showed that boys (Male, β = 0.09, *p* < .01), students with lower prior academic achievement (Repeat, β = 0.03, *p* < .01), and students with less educated mothers (MoEdu, β = -0.01, *p* < .05) reported higher levels of IT integration in their mathematics lessons. On the other hand, students in schools with more full-time (*ln*FTTr, γ = -0.02, *p* < .05) or part-time (*ln*PTTr, γ = -0.02, *p* < .01) mathematics teachers reported lower levels of IT integration. In contrast, average class size was not significantly related to the level of IT integration (ClassSize, γ = 0.00, *p* = .40). The set of control variables explained 0.55% and 3.12% of the level 1 and 2 variances in IT integration respectively.

In Model 3, all the control variables were significantly related to IT integration. More specifically, boys (Male, β = 0.09, *p* < .01), students with lower prior academic achievement (Repeat, β = 0.04, *p* < .01), students with less educated mothers (MoEdu, β = -0.01, *p* < .05), and students in schools with larger average class sizes (ClassSize, γ = 0.01, *p* < .05) reported higher levels of IT integration in their mathematics lessons. On the other hand, students in schools with more full-time (*ln*FTTr, γ = -0.02) or part-time (*ln*PTTr, γ = -0.02) mathematics teachers reported lower levels of IT integration, *p* < .01. Turning to the school IT resource variables, students in schools with more computers per student (CompPerStu, γ = 0.01), and with more IT resources available for students’ use (ITMain, β = 0.08) reported higher levels of IT integration, *p* < .01. The control and school IT resource variables explained 0.78% and 9.03% of the level 1 and 2 variances in IT integration respectively. The increase from 3.12% to 9.03% explained variance at level 2 underscored the importance of school IT resource availability in determining the level of IT integration.

In Model 4, the different variables examined thus far, except ClassSize (γ = 0.00, *p* = .10) remained statistically significant (Male, β = 0.09, *p* < .01; Repeat, β = 0.04, *p* < .01; MoEdu, β = -0.01, *p* < .01; *ln*FTTr, γ = -0.02, *p* < .01; *ln*PTTr, γ = -0.02, *p* < .05; CompPerStu, γ = 0.01, *p* < .05; ITMain, β = 0.08, *p* < .01). As for the institutional variables, students in schools with higher levels of IT expectations in the curriculum (Curriculum, γ = 0.03, *p* < .01) reported higher levels of IT integration. On the other hand, school climate (ClimateTr, γ = -0.01, *p* = .38; ClimateStu, γ = -0.01, *p* = .17) and principal-related activities (Academic, γ = 0.01, *p* = .29; React, γ = 0.01, *p* = .34; Monitor, γ = -0.01, *p* = .33) were not significantly related to the levels of IT integration. The proportion of level 1 variance explained by the control, school IT resource, and institutional variables remained almost unchanged (0.79%), while the proportion of level 2 variance explained increased from 9.03% to 10.56%.

In Model 5, the pattern of relationships between each of these variables and the levels of IT integration remained unchanged (Male, β = 0.09, *p* < .01; Repeat, β = 0.04, *p* < .01; MoEdu, β = -0.01, *p* < .01; ClassSize, γ = 0.00, *p* = .14; *ln*FTTr, γ = -0.02, *p* < .05; *ln*PTTr, γ = -0.02, *p* < .01; CompPerStu, γ = 0.01, *p* < .05; ITMain, β = 0.08, *p* < .01; Curriculum, γ = 0.03, *p* < .01; ClimateTr, γ = -0.01, *p* = .23; ClimateStu, γ = -0.01, *p* = .34; Academic, γ = 0.01, *p* = .11; React, γ = 0.00, *p* = .47; Monitor, γ = -0.01, *p* = .38). With regards to the accountability variables, students in schools with higher levels of parental pressure for academic achievement (ParentExp, γ = -0.02, *p* < .05) reported lower levels of IT integration. However, schools’ public posting of achievement results (DataPublic, γ = -0.01, *p* = .22) or tracking of schools’ achievement results by administrative authorities (DataTracked, γ = 0.02, *p* = .13) was not significantly related to levels of IT integration. The proportion of level 1 variance explained by the control, school IT resource, institutional, and accountability variables remained the same (0.79%), while the proportion of level 2 variance explained increased marginally from 10.56% to 10.92%.

In Model 6, the pattern of relationships between each of these variables and the levels of IT integration remained unchanged (Male, β = 0.09, *p* < .01; Repeat, β = 0.04, *p* < .01; MoEdu, β = -0.01, *p* < .01; ClassSize, γ = 0.00, *p* = .15; *ln*FTTr, γ = -0.02, *p* < .05; *ln*PTTr, γ = -0.02, *p* < .01; CompPerStu, γ = 0.01, *p* < .05; ITMain, β = 0.08, *p* < .01; Curriculum, γ = 0.03, *p* < .01; ClimateTr, γ = -0.02, *p* = .18; ClimateStu, γ = -0.01, *p* = .31; Academic, γ = 0.01, *p* = .16; React, γ = 0.00, *p* = .52; Monitor, γ = -0.01, *p* = .29; ParentExp, γ = -0.02, *p* < .05; DataPublic, γ = -0.01, *p* = .22; DataTracked, γ = 0.02, *p* = .16). One of the two mathematics subject culture variables measuring the presence of school policy on how to use computers in mathematics instruction, Policy, was positively related to levels of IT integration (γ = 0.04, *p* < .01). However, the other variable measuring the degree of pedagogical and curricular differentiation in mathematics lessons (Differentiate) was not significantly related to levels of IT integration (γ = 0.01, *p* = .27). The proportion of level 1 variance explained by the variables remained the same (0.79%), while the proportion of level 2 variance explained increased from 10.79% to 11.45%.

In the last model (Model 7), the pattern of relationships between each of these variables and the levels of IT integration remained unchanged for all the variables except for ClimateTr. More specifically, boys (Male, β = 0.08, *p* < .01), students with less educated mothers (MoEdu, β = -0.01, *p* < .05), and students with lower levels of prior academic achievement (Repeat, β = 0.04, *p* < .01) reported higher levels of IT integration. Additionally, students in schools with more computers per student (CompPerStu, γ = 0.01, *p* < .05), with more access to IT resources (ITMain, β = 0.07, *p* < .01), with higher levels of IT curricular expectations (Curriculum, γ = 0.03, *p* < .01), and with an explicit policy on the use of IT in mathematics (Policy, γ = 0.04, *p* < .01) reported higher levels of IT integration. On the other hand, students who studied in schools with more full- and part-time mathematics teachers (*ln*FTTr, γ = -0.01, *p* < .05; *ln*PTTr, γ = -0.02, *p* < .01 respectively), with more positive school learning climate attributable to teacher-related factors (ClimateTr, γ = -0.03, *p* < .05), and with more academically demanding parents (ParentExp, γ = -0.02, *p* < .01) reported lower levels of IT integration. Class sizes (ClassSize, γ = 0.00, *p* = .30), school learning climate attributable to student factors (ClimateStu, γ = -0.01, *p* = .17), principal leadership (Academic, γ = 0.01, *p* = .39; React, γ = 0.00, *p* = .47; Monitor, γ = -0.01, *p* = .35), schools’ public posting of achievement data (DataPublic, γ = -0.01, *p* = .28), tracking of school’s achievement data by administrative authorities (DataTracked, γ = 0.01, *p* = .25), and pedagogical and curricular differentiation in mathematics lessons (Differentiate γ = 0.00, *p* = .68) were all not related to levels of IT integration. The last set of variables entered into the model pertaining to mathematics teachers’ beliefs and practices were all significantly related to levels of IT integration. More specifically, students whose teachers believed in student-centered teaching-learning (StuFocus, γ = 0.03, *p* < .05) and whose teachers provided more problem-solving activities in class (ProbSolve, β = 0.05, *p* < .01; ProbGive, β = 0.07, *p* < .01) reported higher levels of IT integration. The proportion of level 1 variance explained by the variables increased from 0.79% to 2.24%, while the proportion of level 2 variance explained increased from 11.45% to 15.90, indicating that teacher beliefs and practices were important predictors of IT integration.

## Discussion

The present study employs HLM to (a) examine different IT and non-IT related predictors of the levels of IT integration in schools, and (b) identify which sets of predictors are more important than others in predicting IT integration in school mathematics lessons. IT integration in mathematics lessons was measured using students’ responses to a series of questions asking them if IT had been used in teachers’ demonstration or for students’ learning in mathematics lessons, with responses on students’ involvement taken to be indicative of higher levels of IT integration.

Results showed that students in schools with more computers per student, with more IT resources, with higher levels of IT curricular expectations, with an explicit policy on the use of IT in mathematics, whose teachers believed in student-centered teaching-learning, and whose teachers provided more problem-solving activities in class reported higher levels of IT integration. On the other hand, students who studied in schools with more positive teacher-related school learning climate, and with more academically demanding parents reported lower levels of IT integration. See Table 4 for a summary of the results.

**Table 4:** Summary of Results.

Higher levels of IT integration	Lower levels of IT integration	No significant relationship
• Male students	• Students in schools with more mathematics teachers	• Average class size
• Students with lower prior academic achievement	• Students in schools with more positive teacher-related climate	• Student-related climate
• Students with less educated mothers	• Schools with higher levels of parental pressure for academic achievement	• Principal-related activities
• Schools with more computers per student		• Schools’ public posting of achievement results
• Schools with more availability of IT resources		• Tracking of schools’ achievement results by administrative authorities
• Schools with higher levels of IT expectations in the curriculum		• Degree of pedagogical and curricular differentiation in mathematics lessons
• Presence of school policy on how to use computers in mathematics instruction		
• Teachers who believed in student-centered learning		
• Teachers who provided more problem-solving activities in class		

*Note*. IT integration was measured using students’ responses to seven questions asking them if computers were used in mathematics lessons (1 = *No*, 2 = *Yes*, *but only with teacher demonstration*, 3 = *Yes*, *with students using computers*). A greater value for this variable is viewed as representing a higher level of IT integration.

Put together, the predictors (including the controls) explained a total of 15.90% of the school-level variance in levels of IT integration. Among the variables, access to school IT resources (5.91%) and teachers’ pedagogical beliefs and practices (4.45%) account for a larger proportion of the between-school IT integration variance, as compared to IT curricular expectations (1.53%), parents’ expectations (0.36%), or the presence of IT policies in schools (0.53%).

The usefulness of the myriad variables at the student, subject, and school levels in predicting IT integration underscores the need for policymakers and school leaders to have a comprehensive approach to promote IT-enabled teaching-learning. This plan may include a shared vision and IT integration plan; and strategies to address resource shortages, change teachers’ pedagogical beliefs and practices, provide teachers with professional development opportunities, and include higher-order thinking skills that are susceptible to IT-enabled teaching in assessments [14].

### Access to IT resources

Results showed that variables measuring the access to IT resources in schools constituted the most important set of predictors of IT integration. This finding is consonant with Fabry and Higgs’ [8] contention that IT integration in schools is contingent upon teachers and students’ unfettered access to adequate and appropriate hardware and software. There are many creative ways to increase the access to IT in schools, including schools adopting cheaper computer systems, integrating IT into one or two subject areas at any one time, using laptops equipped with wireless connections instead of computer laboratories, locating computers in classrooms instead of centralized venues, and rotating students in groups through small number of computers in classrooms [45, 46, 47].

### Teachers’ pedagogical beliefs and practices

However, when juxtaposed against the contributions of teachers’ student-centered pedagogical beliefs and practices, IT access appears to be necessary albeit insufficient for IT integration [48]. Indeed, the results showed that teacher variables such as their beliefs in student-centered teaching-learning and the implementation of problem-solving pedagogies constitute the second most important set of predictors of IT integration in terms of the proportion of variance explained. This finding is consistent with those reported in previous studies underscoring the importance of perceived compatibility between teachers’ pedagogical beliefs and the capabilities of IT [16, 34]. It also alludes to teachers using IT for higher-level learning objectives (i.e., providing students with educational opportunities such as problem-solving) instead of more mundane replacement (using IT as an alternative means to teach the same instructional goals) or amplification purposes (using IT to increase effectiveness and efficiency of teaching) [49]. Given the importance of teachers in IT integration, schools can articulate a common vision and strategic plans with regards to integrating IT, provide necessary resources to support teachers, provide teachers with continuous professional development, and provide mechanisms to encourage teachers to experiment with IT integration [50, 51, 52, 53].

### Compensatory use of IT

Results showed negative relationships between IT integration and mothers’ education, and students’ prior academic achievement. Students with less educated mothers and students with lower levels of prior academic achievement reported higher levels of IT integration. These results suggest that schools may have used IT to compensate for the lower learning ability of students from disadvantaged backgrounds (e.g., with lower mothers’ educational attainment). More specifically, results from the present study suggests that some teachers may have used IT integrated lessons more as a remedial or compensatory strategy to address the specific learning needs of lower achieving and unmotivated students. Teachers may have assigned individual use of computer and educational software to lower achieving students more frequently so that students can obtain more immediate and direct learning feedback [54]. This increased usage of IT for compensatory purposes may therefore explain the negative IT-achievement relationships found by other researchers [54].

Contrary to expectations, the results showed that students who studied in schools with less positive teacher-related school learning climate (e.g., teachers being ill-prepared for classes) reported higher levels of IT integration. The reason for this is unclear, although it is probable that ill-prepared teachers might use technology as a babysitter tool for their students, as Green [55] remarked:

“Some teachers want a competent babysitter so they can take break…they fervently wish for a computer lab staffed with a full-time aide [and individual computers] who will receive their students at the door and return to them safe-and-sound 30 minutes later.”

The results also indicated that students in schools with more mathematics teachers and with higher parental pressure for academic achievement reported lower levels of IT integration. Schools with more teachers may find it difficult to schedule computer labs for individual students to use. The parental pressure for students to score high on tests would create a daunting challenge for teachers. Teachers probably feel that they can teach more content when they used technology to didactically present or demonstrate the mathematics topics, rather than allowing students to use computers on their own to explore the topics because of the additional time required for the latter.

### Insignificant relationships

The results also showed that there was no significant relationship between IT integration and the following variables: average class size, student-related school learning climate, principal-related activities, schools’ public posting of achievement data, tracking of school’s achievement data by administrative authorities, and pedagogical and curricular differentiation in mathematics lessons. The Pearson correlations between these variables, except principals’ monitoring and schools’ pedagogical/curricular differentiation, and IT integration were significant, but they became insignificant in the HLM models.

The non-significant relationship between average class size and student use of technology is consistent with the findings of Ritzhaupt and colleagues [42]. It seems that student to computer ratio is a more important predictor variable of student technology use compared to mere class size.

It is interesting that only the teacher- but not student-related aspects of school learning climate were related to IT integration in schools. This finding is consistent with the literature highlighting that teacher characteristics, more so than student or other school attributes, may be more instrumental to the use of IT in teaching [16, 34]. It also underscores the agency of teachers in addressing challenges of unmotivated students–they can decide to use IT or other means to enhance learning.

The finding that different principal leadership behaviors were not predictive of IT integration alludes to the indirect, mediated effects that school leaders have on school and student outcomes [56]. The school effectiveness literature has found that principals impact student achievement indirectly by developing the school’s instructional capacity (setting school vision; supporting and monitoring teaching processes; and building systems, structures, and processes) so that teachers at the frontline of teaching are better supported to teach effectively [57]. Therefore, it is likely that principals influence IT integration in teaching through other proximal processes such as influencing teachers’ beliefs on the use of IT and providing teachers and students with access to IT as the results of our study have shown.

The results also showed that schools’ public posting of achievement data or the tracking of school’s achievement data by administrative authorities was not significantly related to IT integration. These results contrasted with that for parental expectations which was found to negatively predict IT integration. Given the neoliberal context that many schools are operating in [58, 59], it is understandable that parents, being stakeholders who are most proximal to schools, are more influential than more remote school inspections (e.g., via monitoring of results) or the larger community-at-large (e.g., who evaluate school performance via the pubic posting of students’ results) [59, 60, 61]. In any case, external monitoring of school performance by educational authorities may also be influenced by parental expectations.

The reason for the non-significant finding between pedagogical/curricular differentiation in mathematics lessons and IT integration is unclear. On the one hand, it can be reasoned that teachers may employ IT to cater to students’ diverse learning needs in differentiation [49]. On the other hand, teachers have at their disposal many other different platforms and strategies to choose from in customizing their teaching [62]. Therefore, future research may investigate how teachers cater to diverse students’ learning needs using IT or other means.

In sum, it is perhaps premature to definitively conclude that the aforementioned variables are not related to IT integration as some of them may be *indirectly* related to IT integration (e.g., principal leadership). Therefore, future research can examine how the myriad variables, significant or otherwise, are related to each other (e.g., the relationship among schools’ public posting of results, external monitoring, and parental expectations), and the direct and indirect effects they have on IT integration [14].

## Conclusion

The present study examined what predicted IT integration in secondary school mathematics lessons. Data from 32,256 secondary school students from 2,519 schools in 16 developed economies who participated in PISA 2012 were analyzed. For the purposes of the present study, IT integration was defined as the use of computing devices such as desktop computers, laptops, tablets, software, or Internet for learning mathematics in schools. It is noteworthy that IT integration was measured using students’, instead of teachers’, reported data, thereby circumventing the problems of possible teachers’ inaccurate responses or social desirability bias [43].

A variety of independent variables was examined for their relationship with IT integration. The availability of the multitudinous variables allows for the examination of the independent effects of each predictor after controlling for other predictor and student- and school-level control variables. This approach also represents a significant methodological improvement over isolated studies focusing on specific variables [16, 18]. To manage the nested data, the present study employed HLM to address the possible correlation in achievement scores of students belonging to the same school and to partition achievement into between-school (the appropriate level for the purposes of the present study) as opposed to within-school variance [20].

Results showed that after controlling for student-level (gender, prior academic achievement and socioeconomic status) and school-level (class size, number of mathematics teachers) variables, students in schools with more computers per student, with more IT resources, with higher levels of IT curricular expectations, with an explicit policy on the use of IT in mathematics, whose teachers believed in student-centered teaching-learning, and whose teachers provided more problem-solving activities in class reported higher levels of IT integration. On the other hand, students who studied in schools with more positive teacher-related school learning climate, and with more academically demanding parents reported lower levels of IT integration. Student-related school learning climate, schools’ public posting of achievement data, tracking of school’s achievement data by administrative authorities, and pedagogical and curricular differentiation in mathematics lessons were not related to levels of IT integration. Put together, the predictors explained a total of 15.90% of the school-level variance in levels of IT integration. In particular, school IT resource availability, and mathematics teachers’ pedagogical beliefs and practices stood out as important determinants of IT integration in mathematics lessons.

The present study contributes to the literature in two ways. First, it provides evidence on the myriad IT and non-IT related variables that may predict levels of IT integration in mathematics lessons. The results address the knowledge gap arising from the bias on investigating proximal IT-related variables in previous studies. They also underscore the relative importance of teachers’ pedagogical beliefs and practices, and access to school IT resources by teachers and students in IT integration as compared to other school contextual/institutional or mathematics subject culture variables. The second contribution is the insights on the plausible compensatory use of IT for lower-achieving students who may be studying in poorly resourced schools. These insights add to the evidence pointing to negative association between IT and students’ achievement reported in some studies.

Notwithstanding these contributions, the present study is unable to provide conclusive causal claims regarding what predictors contribute to IT integration because of its cross-sectional analysis. Also, while the focus on developed economies in the present study enables meaningful comparisons to be made, a case can be made that different variables may predict IT integration in less-developed economies. Future research may employ multi-level structural equation modelling to examine the relationships among different predictor variables and IT integration in schools. In particular, this analytical approach may unravel indirect effects on IT integration for variables that are found to be insignificant in the present study. Longitudinal or experimental research designs will also address questions of causality. The knowledge base will also benefit from future research examining the differential impact of IT integration on students of different SES and prior academic achievement profiles.
